# The potential of *Panaxnotoginseng* against COVID-19 infection

**DOI:** 10.1016/j.jgr.2023.04.002

**Published:** 2023-04-08

**Authors:** Yeye Hu, Ziliang He, Wei Zhang, Zhiqiang Niu, Yanting Wang, Ji Zhang, Ting Shen, Hong Cheng, Weicheng Hu

**Affiliations:** a Institute of Translational Medicine, School of Medicine, Yangzhou University, Yangzhou, China; b School of Life Sciences, Huaiyin Normal University, Huaian, China; c Affiliated Hospital of Yangzhou University, Yangzhou, China; d Jiangsu Key Laboratory of Experimental & Translational Non-Coding RNA Research, School of Medicine, Yangzhou University, Yangzhou, China

**Keywords:** *Panax notoginseng*, COVID-19, cytokine storm, potential therapeutics

## Abstract

The COVID-19 pandemic has changed the world and has presented the scientific community with unprecedented challenges. Infection is associated with overproduction of proinflammatory cytokines secondary to hyperactivation of the innate immune response, inducing a cytokine storm and triggering multiorgan failure and significant morbidity/mortality. No specific treatment is yet available. For thousands of years, *Panax notoginseng* has been used to treat various infectious diseases. Experimental evidence of *P.* notoginseng utility in terms of alleviating the cytokine storm, especially the cascade, and improving post-COVID-19 symptoms, suggests that *P.* notoginseng may serve as a valuable adjunct treatment for COVID-19 infection.

## 1 Introduction

The novel coronavirus disease termed “COVID-19” by the World Health Organisation (WHO) is an acute respiratory disease caused by the severe acute respiratory syndrome coronavirus 2 (SARS-CoV-2). The virus is highly contagious and has triggered a rapidly spreading worldwide epidemic associated with severe economic and social losses [1,2]. SARS-CoV-2 infection induces an abnormal immune response, characterised by excessive release of proinflammatory cytokines [3]. There is as yet no specific treatment. Vaccines are the most economical and effective means by which to control viral infections, but may afford inadequate protection, are expensive, and take time to design and produce. Also, it is difficult to identify/predict the viral strains against which protection is required [4]. Side-effects and rapid emergence of drug-resistance limit the utilities of existing drugs [5]. Antivirals that trigger protective immune responses and inhibit viral replication are needed.

Many herbal and plant extracts exert antiviral effects [[6], [7], [8]], affording many opportunities for the development of new drugs that are highly efficient, minimally toxic, and exert few side-effects. Numerous studies have found that herbal medicines greatly enhance immunity, improve health, and reduce the severity of COVID-19 symptoms [[9], [10], [11]]. The bioactivities of *Panax notoginseng* are similar to those of the more widely known *Panax* ginseng [12]. *P.* notoginseng is widely used to prevent and treat various conditions. The earliest scientific description of *P.* notoginseng in Compendium of Materia Medica recorded that *P.* notoginseng alleviate pain caused by blood disease. Scientific studies indicated that *P.* notoginseng possessed multiple pharmacological activities including antioxidant [13], anti-inflammatory and antimicrobial [14], hypolipidemic [15], hepatoprotective, antitumor [16], anti-atherosclerotic, and neuroprotective effects [[17], [18], [19]]; *P.* notoginseng also regulates the immune system and may improve health by regulating the immune and inflammatory responses of various pathological scenarios including viral infection. According to Chinese Medicine Dictionary and China Pharmacopoeia, *P.* notoginseng has been incorporated into several preparations for treatment of cardiovascular disease, inflammation, and body pains [20,21]. The formula containing *P.* notoginseng was recommended to combat the novel coronavirus pneumonia caused by this fast-spreading virus COVID-19 in Wuhan, China. Therefore, here, we discuss the potential effects of *P.* notoginseng against COVID-19 infections, which including antiviral activity, enhancement of immunity, and suppression of the inflammatory cytokine storm triggered by excessive innate immunity.

## 2 Potential of *P.* notoginseng to protect against SARS-CoV-2

SARS-CoV-2 infection disrupts normal immune responses, compromising the immune system and triggering uncontrolled inflammatory responses in patients with severe/critical COVID-19 illness. The swift emergence of new viral variants limits the effectiveness of antiviral drugs and vaccines. Management of the SARS-CoV-2 immune response in a manner that enhances antiviral immunity and suppresses systemic inflammation may be the key to successful treatment.

According to Sun et al [22], *P.* notoginseng extract (PNE) supplementation significantly increased growth and enhanced immunity in hybrid grouper fish fed high-lipid diets. Dietary PNE increased the expression levels of antioxidant- and immune system-related genes, and anti-inflammatory cytokines; the optimal PNE dose was 0.5 g/kg in farmed fish. The antiviral activities of *P.* notoginseng are attributable to enhanced host immunity. In mice exposed to influenza A virus (H1N1), *P.* notoginseng root (PNR) water extracts reduced mortality by 90% and protected against weight loss (compared to controls). Spleen cells from PNR-treated mice exhibited increased NK cell activity against YAC-1 cells [23]. The innate immune system is the first line of defense against viral infection. Thus, NK cells play important roles in such early defense [24,25]. Inhibition (or removal) of mouse NK cells triggers morbidity and mortality, and delays viral clearance [26]. Choi et al [23] suggested that PNR stimulated a dose-dependent antiviral response in mouse macrophages that significantly protected mice against viral infection, perhaps because PNE stimulated NK cell activity. Macrophages are normally scattered throughout the body, respond rapidly to infection and kill pathogens either directly (via phagocytosis) or indirectly (via secretion of pro-inflammatory mediators). Macrophages inhibit viral replication and prevent cancer, and *P.* notoginseng improves resistance to viral infections and cancer. Rhule et al [27] found that *P.* notoginseng exerted immunomodulatory effects on cultured macrophages. PNR pretreatment suppressed viral replication in RAW264.7 cells and inhibited the expression of the viral proteins PB1, PB2, HA, NA, M1, PA, M2 and NP; and that of viral mRNAs encoding NS1, HA, PB2, PA, NP, M1 and M2 [23]. Immune destruction evasion is an emerging feature of cancer; PNR served as a tumoricidal effector by redirecting macrophages. Water extracts limited M2 activation but stimulated M1 activation [28]. Dendritic cells (DCs) (which link the innate and adaptive immune systems) play central roles in modulating inflammation and adaptive immunity. Rhule et al were the first to describe the immunomodulatory effects of *P.* notoginseng on several TLR ligands of mouse DCs; after toll-like receptor activation, *P.* notoginseng inhibited secretion of specific inflammatory cytokines and expression of the innate immune responses [29]. Together, the data show that *P.* notoginseng reduces the inflammatory responses of DCs to bacteria or viruses.

**Fig. 1:**
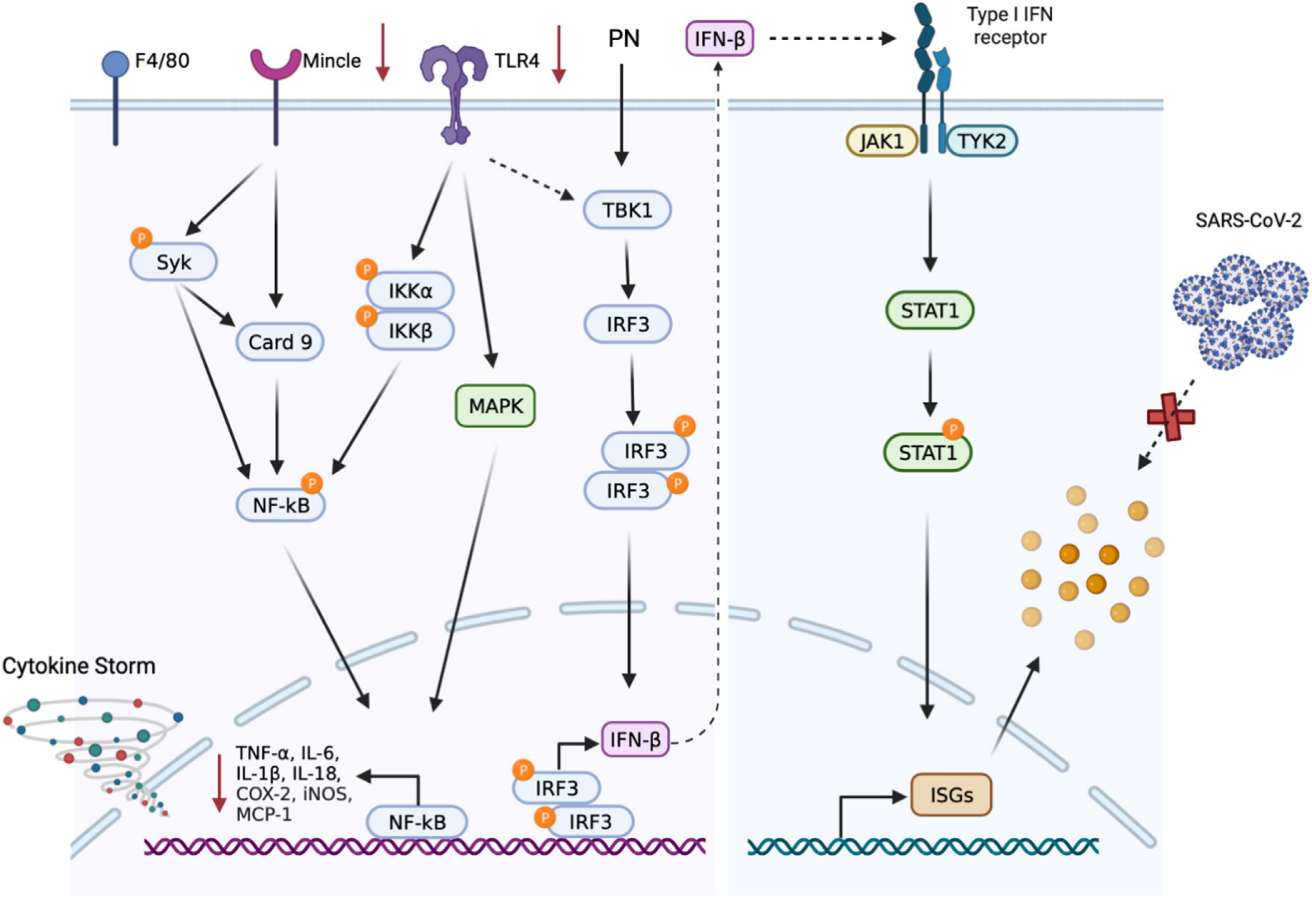
This schematic diagram illustrates the mechanisms of the potential of *Panax notoginseng* against COVID-19 infection.

An excessive immune response produces large amounts of pro-inflammatory cytokines (TNF-α, IL-1β, IL-6, IL-18 and others) that maintain the abnormal systemic inflammatory response, which not only removes pathogenic microorganisms but also attacks the body, triggering multiple organ failure [30,31]. After COVID-19 infection, cytokine levels are elevated [32]. Many reports have suggested that a “cytokine storm” (uncontrolled cytokine overproduction) is a major cause of immune system pathogenesis in such patients [[33], [34], [35], [36]]. Interferons, interleukins, chemokines, and tumor necrosis factors are all involved in development of the cytokine storm; IL-6, IL-1β, IL-8, IL-10 and TNF-α are of particular importance in this context [37,38]. Huang et al [39] reported that the plasma levels of the inflammatory cytokines IL-2, IL-7, IL-10, IFN-γ, MCP-1 and TNF-α in intensive care unit (ICU) patients were higher than in non-ICU patients. Recent studies have shown that severely ill patients had higher IL-6 levels than those with mild and moderate illness [40]. The anti-inflammatory effects of *P.* notoginseng are widely known. *P.* notoginseng inhibited cytokine expression (of all of TNF-α, IL-1β, and IL-6) by macrophages, thus exerting anti-inflammatory and immunosuppressive properties [27]. Jung et al suggested that the strong anti-inflammatory properties of *P.* notoginseng flower (PN–F) reflected inhibition of both NF-κB activation and the expression of inflammation-related genes (encoding iNOS, COX-2, TNF-α, and IL-1β) [41]. The anti-inflammatory effects of a methanol extract of *P.* notoginseng on LPS-induced RAW264.7 cells were stronger than those of a water extract [42]. In contrast, raw *P.* notoginseng afforded better anti-inflammatory effects but steamed *P.* notoginseng better antioxidant and haematopoietic effects, consistent with “the raw eliminate and the steamed tonify” view [43,44]. Thus, *P.* notoginseng regulates various aspects of inflammation *in vitro* and also inflammatory diseases *in vivo*. Sepsis is caused by bacteria and toxins that hyperactivate the systemic inflammatory response [45]. Shou et al established a septic acute kidney injury (AKI) model in male SD rats (via cecal ligation and puncture); *P.* notoginseng powder (PNP) reduced the levels of IL-18, IL-1β, TNF-α and IL-6, substantially ameliorating the inflammatory response [46]. Rheumatoid arthritis (RA) is an inflammatory autoimmune disease of joints. In a model of collagen-induced arthritis (CIA), the disease-modifying effects of BT-201 (an n-butanol extract of *P.* notoginseng) suggested that the extract might usefully augment anti-TNF-α treatment of inflammatory diseases [47]. Chronic colonic inflammation may trigger cancer [48]. Wen et al showed that *P.* notoginseng exerted anti-inflammatory actions in an mouse model of experimental colitis induced by azoxymethane (AOM)/dextran sulfate sodium (DSS) [49]. In summary, the data suggest that *P.* notoginseng may reduce inflammation caused by SARS-CoV-2 (Table 1, Fig. 1).

**Table 1:** *Panax notoginseng* as Potential Therapeutic Agents for COVID-19

Names	Models	Inflammatory modulators	Signaling pathways	Effects	References
*Panax notoginseng*	AOM/DSS mouse model	(−) Enzymes (iNOS and COX-2)		Anti-inflammatory	[49]
	LPS-induced RAW264.7 macrophages	(−) Cytokine (TNF-α); (−) mediator (NO)		Anti-inflammatory	[42]
	LPS-stimulated RAW264.7 cells	(−) Cytokines (TNF-α and IL-1β); (−) enzymes (iNOS and COX-2); (−) mediators (NO and PGE2)	MAPK; NF-κB	Anti-inflammatory	[41]
	LPS-induced RAW264.7 cells	(−) Cytokines (TNF-α, IL-6, and IL-1β); (−) enzyme (COX-2); (−) costimulatory molecules (CD40 and CD86)		Immunomodulatory	[27]
	Septic AKI model	(−) Cytokines (IL-18, IL-1β, TNF-α, and IL-6)	NF-κB	Anti-inflammatory	[46]
	LPS-stimulated THP-1 cells	(−) Cytokine (TNF-α)	MAPK; NF-κB	Anti-inflammatory	[47]
	PMA-stimulated THP-1 cells	(−) Cytokine (IL-1β)			
	LPS-stimulated RAW264.7 cells	(−) Mediator (iNO)			
	TNF-α-induced SW1353 cells	(−) Enzyme (MMP-13)			
	CIA model				
	Ear edema model			Anti-inflammatory	[43]
	LPS-induced RAW264.7 cells	(−) Cytokines (TNF-α and IL-6)		Anti-inflammatory	[44]
	LPS-induced DC2.4 cells	(−) Cytokines (TNF-α and IL-6); (−) costimulatory molecules (CD40 and CD86)		Immunomodulatory	[29]
	TLR ligand-induced DC2.4 cells				
	RAW264.7 cells	Activate M1 phenotype macrophage		Immune	[28]
	Tumor allograft model				
APF	LPS-induced BMDM cells	(−) Cytokines (TNF-α, IL-6, and IL-1β); (−) enzyme (iNOS); (−) chemokine (MCP-1); inhibit M1and activate M2 macrophages	Mincle/Syk/NF-κB	Anti-inflammatory; immune	[50]
	Cisplatin-induced AKI				
	5/6 nephrectomy induced CKD mouse model	(−) Cytokines (TNF-α, IL-6, and IL-1β); (−) chemokine (MCP-1); (−) enzyme (iNOS); inhibit M1and activate M2 macrophages	Mincle/NF-κB	Anti-inflammatory; immune	[51]
	LPS and indophenol sulfate induced RAW264.7 cells	(−) Cytokines (TNF-α, IL-6, and IL-1β); (−) enzyme (iNOS); (−) chemokine (MCP-1)			
	High-fat and high-sugar diet and streptozotocin established diabetic nephropathy model	(−) Cytokines (TNF-α, IL-6, and IL-1β)	Mincle/Card9/NF-κB	Anti-inflammatory; immune	[52]
	High-glucose induced BMDM cells				
	High-glucose induced BMDM and MES cells

The *Astragalus mongholicus* Bunge and *P.* notoginseng formula (APF) is a widely used traditional medicine for the treatment of chronic kidney inflammation. In a model of cisplatin-induced acute kidney injury, APF significantly reduced the levels of IL-1β, IL-6, TNF-α and MCP-1 by inhibiting the mincle/Syk/NF-κB signaling pathway. Also, APF reduced activation of pro-inflammatory M1 macrophages and increased that of anti-inflammatory M2 macrophages [50]. The effects of a combination of APF and *Bifidobacterium* were consistent with these results [51]. Lin et al found that APF improved renal function and inflammation in a model of diabetic nephropathy by inhibiting the Mincle/Card9/NF-κB signaling pathway [52]. In summary, the traditional Chinese medicine (TCM) formula APF inhibits the inflammatory responses of macrophages and may thus usefully treat COVID-19 infection.

## 3 Potential of *P.* notoginseng relieve post-COVID-19 symptom burden

A growing body of research documents that the patients experienced increased myalgia, anxiety, extreme fatigue, low mood, and sleep disturbance during the post-COVID-19 period [[53], [54], [55]]. *P.* notoginseng is generally used as a remedy to enhance stamina, relieve anxiety, combat stress, alleviate fatigue, reduce pain and swelling [[56], [57], [58]]. Liang et al [59] investigated and concluded that PNG supplement improved endurance time to exhaustion and lowered mean blood pressure (MAP), enhancing physical performance during endurance exercise. In a double-blind randomized placebo-controlled trial, the use of *P.* notoginseng exhibited positive trends in performance and pain following delayed onset muscle soreness (DOMS) inducing exercise [60]. Moreover, orally administered *P*. notoginseng root dry extract regulated emotional responses in rats [61]. Li et al also surveyed most recent 20 years of research on *P*. notoginseng for treating depression, which has been shown to have a therapeutic effect on depression [62].

## 4 Conclusions

The COVID-19 pandemic has greatly damaged human health and has posed unprecedented challenges. Unfortunately, there is currently no proven therapeutic intervention countering the (potentially) life-threatening cytokine storm caused by SARS-CoV-2. Here, we have discussed the pharmacological potential of *P.* notoginseng; the material may control the cytokine storm. Accumulating evidence points to an anti-viral potential of *P.* notoginseng both *in vitro* and *in vivo*. In China, TCM formulae including Lianhuaqingwen, Jinhuaqinggan and Xuebijing are used to treat COVID-19 infection; natural products may be valuable in this context. *P.* notoginseng, a representative herbal medicine, and the various extracts thereof and mixed *P.* notoginseng compounds, inhibit the actions of proinflammatory cytokines (IL-1β, IL-6 and TNF-α) by modulating signaling pathways including the MAPK, Mincle/NF-κB and JAK/STAT pathways. In addition, *P*. notoginseng can also relieve post-COVID-19 symptom. However, there is no direct evidence that natural products significantly assist COVID-19 patients. Therefore, we focused on whether *P.* notoginseng might be a useful (future) adjuvant treatment for COVID-19 infection. More preclinical and clinical trials are required before *P.* notoginseng can be safely used to quell the cytokine storm of COVID-19 infection.
